# Data for the morphometric characterization of NT2-derived postmitotic neurons

**DOI:** 10.1016/j.dib.2016.04.021

**Published:** 2016-04-13

**Authors:** Imanol González-Burguera, Ana Ricobaraza, Xabier Aretxabala, Sergio Barrondo, Gontzal García del Caño, Maider López de Jesús, Joan Sallés

**Affiliations:** a Department of Pharmacology, Faculty of Pharmacy, University of the Basque Country (UPV/EHU), Paseo de la Universidad 7, 01006 Vitoria-Gasteiz, Araba, Spain; b Department of Neurosciences, Faculty of Pharmacy, University of the Basque Country (UPV/EHU), Paseo de la Universidad 7, 01006 Vitoria-Gasteiz, Araba, Spain; c CIBERSAM, Spain

## Abstract

NTERA2/D1 human teratocarcinoma progenitors induced to differentiate into postmitotic neurons by either long-term treatment with retinoic acid or short-term treatment with the nucleoside analog cytosine β-D-arabinofuranoside were subjected to morphometric analysis and compared. Our data provide a methodological and conceptual framework for future investigations aiming at distinguishing neuronal phenotypes on the basis of morphometric analysis. Data presented here are related to research concurrently published in “Highly Efficient Generation of Glutamatergic/Cholinergic NT2-Derived Postmitotic Human Neurons by Short-Term treatment with the Nucleoside Analogue Cytosine β-D-Arabinofuranoside” [1].

**Specifications Table**

**Table:** 

Subject area	*Biology*
More specific subject area	*Neuronal differentiation*
Type of data	*Text file, graph, and figure*
How data was acquired	*Epifluorescence microscope for detection of β-III tubulin, NeuN/Fox-3 and Hoechst 33342 signals, NIH ImageJ software for morphometrical analysis, GraphPad Prism 5.0 software for statistical analysis*
Data format	*Processed images, analyzed data*
Experimental factors	*NT2 progenitors were induced to differentiate with either retinoic acid (RA) or cytosine β-D-Arabinofuranoside (AraC)*
Experimental features	*Images of immunostained neurons derived from NT2 progenitors were acquired with an epifluorescence microscope and subjected to morphometric analysis*
Data source location	*Department of Pharmacology, Faculty of Pharmacy, University of the Basque Country (UPV/EHU), Spain*
Data accessibility	*Data is provided within the article*

## Value of the data

• The present data describe a reliable methodology to morphometrically distinguish different neuronal phenotypes *in vitro*.
• By combining phenotype markers and morphometric analysis, data provide a methodological approach to differentiate stem cell-derived neuronal and non-neuronal phenotypes.
• These data are useful for researchers interested in stem cell-derived neurons.

## 1. Data

**Fig. 1:**
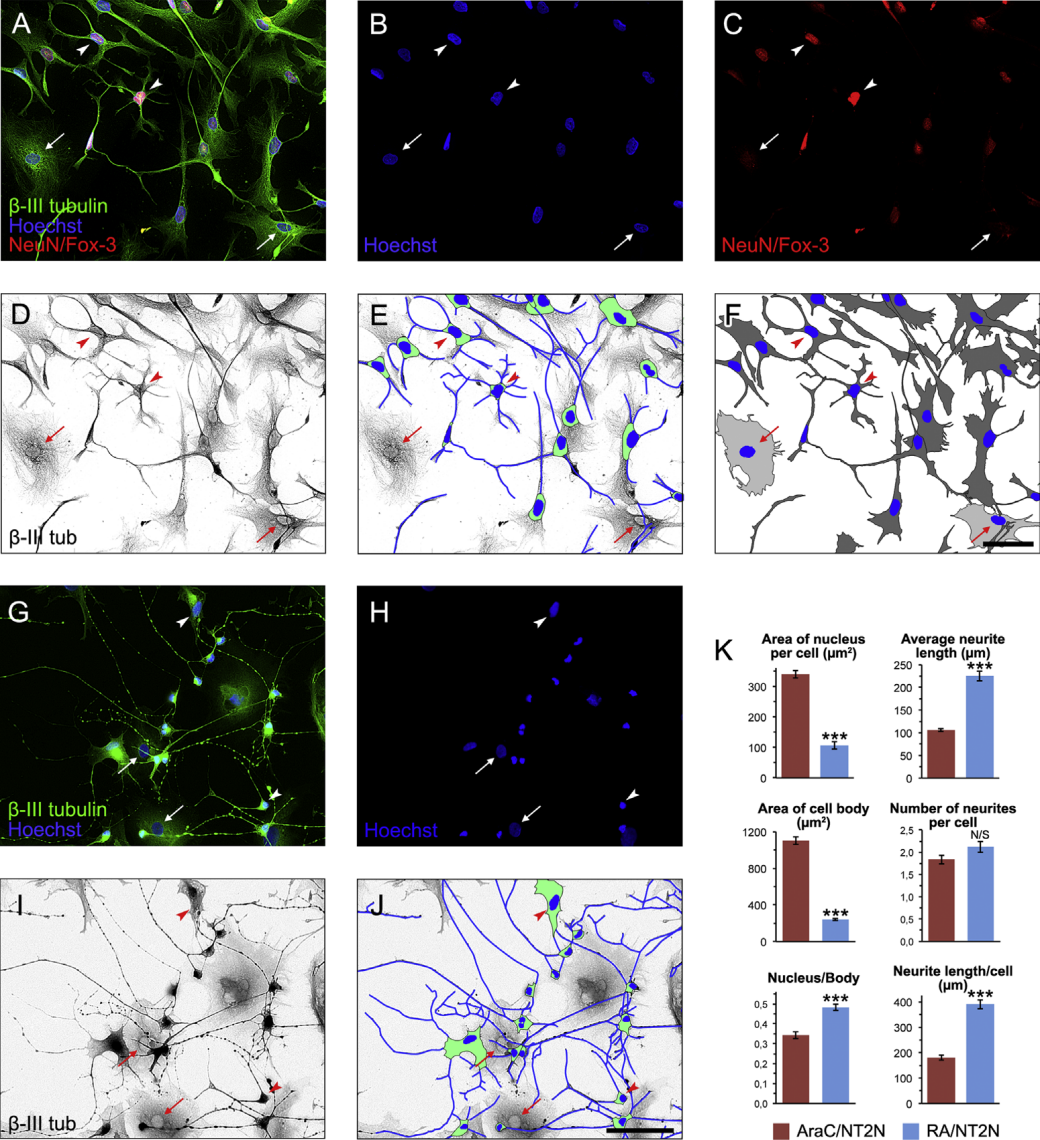
Illustration of image processing for morphometric analysis of NT2N neurons obtained by treatment with 20 µM AraC (AraC/NT2N, A–F) for 6 days or 10 µM RA (RA/NT2N, G–I) for 4 weeks. Mosaic images of cell cultures processed for immunolabeling with β-III tubulin (green) combined with Hoechst’ staining (blue) were captured under either a 20X (A) or a 40X objective (G). Binarized images of Hoechst-stained nuclei (B,H) and inverted grayscale images of β-III tubulin immunostaining (D–I) were used to measure the area of neuronal nuclei and bodies (E,J). In AraC-treated cultures, co-immunostaining for NeuN/Fox-3 (red) and β-III tubulin (A,C) was used to identify non-neuronal (arrows in A–F; light gray-filled cells in F) and neuronal (arrowheads in A–F; dark gray-filled cells in F) phenotypes, which were subjected to additional morphometric analysis with nuclear and whole cell areas as variables (see Materials and methods). In contrast, RA/NT2N neurons (arrowheads in G–J) could be easily distinguished from non-neuronal cells (arrows in G–J) by their size and morphology. Scale bars=100 µm. K. Bar graphs showing values obtained from morphometric analyses. All data represent average values obtained from 99 cells in 3 independent experiments. Two-tailed unpaired *t*-test (Mean±SEM; *n*=3; ***, *p*<0.0001; N/S, not significant).

Pluripotent NTERA2/D1 (NT2) human teratocarcinoma cells induced to differentiate into postmitotic neurons (NT2N neurons) by either short-term treatment (6 days) with cytosine β-D-arabinofuranoside (AraC) [1], [2] (Fig. 1A–F) or long-term treatment (4 weeks) with retinoic acid (RA) [1], [3], [4] (Fig. 1G–J) were morphometrically analyzed. The data obtained were statistically treated and used for comparison between AraC- and RA-differentiated neurons (AraC/NT2N and RA/NT2N neurons, respectively). Mean values (±SEM) for nuclear area, average neurite length per cell, area of the soma, number of neurites per cell, ratio of nuclear area to the body area and total neurite length per cell, resulting from morphometric analysis of AraC/NT2N and RA/NT2N neurons, are shown in Fig. 1K.

## 2. Experimental design, materials and methods

### 2.1. Immunofluorescence labeling

NT2 progenitors were cultured and induced to differentiate into NT2N postmitotic neurons using either cytosine β-D-Arabinofuranoside (AraC) or retinoic acid (RA) as previously described in our laboratory [1], thus obtaining cultures highly enriched in AraC/NT2N or RA/NT2N postmitotic neurons. Because β-III tubulin was expressed in cell bodies and throughout all neurites of differentiated AraC/NT2N and RA/NT2N cells, delivering at the same time high contrast [1], we chose an antibody against β-III tubulin as the primary marker for measurements of area of neuronal body and neurite length in both neuronal phenotypes. Additionally, AraC-treated cultures were co-immunolabeled with an antibody against the neuronal marker NeuN/Fox-3, which served to establish a double criterion classify cells as neuronal or non-neuronal (see below).

For immunofluorescence labeling, cells were fixed, for 10 min at 20–25 °C, with 4% paraformaldehyde in phosphate-buffered saline 0.1 M, pH 7.4 (PBS). After 3 washes (10 min each) at 20–25 °C with washing buffer (PBS, containing 0.22% gelatin, Panreac, Barcelona, Spain, and 1% serum albumin bovine, Sigma-Aldrich, St Louis, MO, USA), cells were incubated at 4 °C for 1 h with permeabilizing blocking buffer (washing buffer, containing 0.05% saponin, Sigma-Aldrich, and 1% normal serum from the species in which the secondary antibody was raised). Thereafter, cells were incubated with primary antibodies diluted with permeabilizing blocking buffer at 4 °C overnight. As primary antibody against β-III tubulin, we used the affinity-purified chicken polyclonal antibody to β-III tubulin (1:1,000; Abcam, ab41489), either alone (for single immunofluorescence) or combined (for double immunofluorescence) with mouse monoclonal to NeuN/Fox-3 (1:1000; Millipore, MAB377, clone A60). After 3 washes (10 min each) at 20–25 °C with washing buffer, cells were incubated with the appropriate fluorescent dye-conjugated secondary antibodies diluted with permeabilizing blocking buffer for 1 h at 20–25 °C. Thus, Dylight 488 Donkey anti-Chicken IgG secondary antibody (1:400; Jackson Immunoresearch Laboratories, Inc.; West Grove, PA, USA, 703-486-155) was used either alone, for single β-III tubulin immunofluorescence, or combined with Alexa Fluor 568 Goat anti-Mouse IgG (Invitrogen Molecular Probes, Spain, A-11031), for double immunofluorescence against β-III tubulin and NeuN/Fox-3. Both secondary antibodies consist of affinity-purified IgGs adsorbed against several species to prevent undesired cross-reactions in double immunolabeling experiments. After the secondary antibody incubation, cells were washed once with washing buffer for 10 min at 20–25 °C. Cell nuclei were then counterstained with Hoechst 33342 (Sigma-Aldrich, diluted to 0.1 µg/mL with washing buffer) for 10 min at 20–25 °C. After 2 additional washes (10 min each) at 20–25 °C with washing buffer, cells were mounted onto glass slides using homemade Mowiol (Calbiochem, Madrid, Spain) mounting medium, containing anti-fade reagent 1,4-phenylenediamine dihydrochloride (Sigma-Aldrich).

### 2.2. Morphometric analyses

RA/NT2N neurons could be easily distinguished from the remaining non-neuronal cells on the basis of their much stronger β-III tubulin immunostaining intensity and morphological features. In contrast, both AraC/NT2N neurons and non-neuronal cells remaining after the 6-day period of treatment with AraC were strongly immunopositive for β-III tubulin and, consequently, could not be distinguished from AraC/NT2N neurons on the basis of the expression of this neuronal marker. Thus, to identify neuronal and non-neuronal cells in cultures treated with AraC for 6 days, absence of or very weak immunolabeling for the neuronal marker NeuN/Fox-3 was used as the first cut-off criterion to classify cells as non-neuronal. Additionally, cells showing weak to moderate immunoreactivity for NeuN/Fox-3 but displaying flat polygonal morphologies and no neurites were also classified as non-neuronal (Fig. 1A–F, arrows), whereas cells showing NeuN/Fox-3 positive nucleus and neurite extensions were classified as AraC/NT2N neurons (Fig. 1A–F, arrowheads). Hence, morphometric analysis of terminally differentiated AraC/NT2N neurons and non-neuronal cells remaining after the 6-day period of treatment with AraC was performed on fluorescence microscope images of cells doubly stained for β-III tubulin and NeuN/Fox-3, and counterstained with Hoechst 33342 (Fig. 1A–F), whereas morphometric analysis of RA/NT2N neurons was performed on fluorescence microscope images of cells immunostained for β-III tubulin and counterstained with Hoechst 33342 (Fig. 1G–J).

Cells were imaged by conventional epifluorescence using a Carl Zeiss Axio Observer.Z1 microscope, equipped with a HXP120C metal halide lamp and a XYZ motorized stage (all from Carl Zeiss MicroImaging, Inc, Gottingen, Germany). Immunolabeled AraC/NT2N and RA/NT2N neurons were imaged using 20x Plan-Apochromat (NA 0.8; pixel size 0.322×0.322 µm^2^) and 40x EC Plan Neofluar (NA 0.75; pixel size 0.161×0.161 µm^2^) objectives (both from Carl Zeiss MicroImaging, Inc), respectively. Bandpass filters used (all from Carl Zeiss MicroImaging, Inc) were 38 HE eGFP (Ex 470/40, Em 525/50) for Dylight 488, 43 HE Cy3 shift free (Ex 550/25, Em 605/70) for Alexa Fluor 568 and 49 DAPI (Ex G 365/Em 445/50) for Hoechst׳s staining. Monochrome images of each color channel were captured with a resolution of 1388×1040 pixels and 16-bit depth using a high-resolution monochromatic camera AxioCam MRm (Carl Zeiss MicroImaging, Inc) and digitized using Zeiss Axio Vision 4.8 software (Carl Zeiss MicroImaging, Inc). The captured 16-bit images were then converted to TIFF files using the ImageJ software (NIH, Bethesda, MA, USA). Multiple images from cultures were taken using the Carl Zeiss Mosaic module with focus adjustment. Subsequent stitching and aligning individual images with an overlap of 10% resulted in 2.0×1.5 mm mosaic images. Measurements were performed on 3 mosaic images obtained from 3 independent experiments. A total of 33 cells of each phenotype (cells classified as non-neuronal in AraC-treated cultures, AraC/NT2N neurons and RA/NT2N neurons) were analyzed per mosaic, starting from the center of the image. Fig. 1A–F and G–J correspond to representative portions of a mosaic images used for morphometric analysis and depict the different steps of image processing to obtain values of the parameters analyzed. To measure the area of nuclei, binary images were obtained by thresholding the Hoechst channel (Fig. 1B and H) to include only nuclei using ImageJ software (NIH). Inverted grayscale images from the β-III tubulin channel (Fig. 1D and I) allowed visualization of cell bodies of AraC/NT2 and RA/NT2 neurons (Fig. 1E and J), as well as the whole cell area of AraC-treated cells, including lamellar extensions of AraC/NT2N neurons (Fig. 1F). The Hoechst channel and inverted grayscale images corresponding to the β-III tubulin channel were loaded as separate image stacks in ImageJ software. Area of nucleus, area of cell body and length of neurites was determined for each AraC/NT2N and RA/NT2N neuron. The area of nuclei was selected (Fig. 1E and J) on the corresponding stack with the wand (tracing) tool of ImageJ and then measured. Unhealthy or fragmented nuclei and false-positive particles were excluded from the analysis. Cell somata were manually outlined and their area measured (Fig. 1E and J). When the boundaries were not clearly defined due to the presence of flat lamellar extensions in AraC/NT2N neurons, β-III tubulin staining accumulation was used to define the limits of neuronal bodies (Fig. 1E). Neurites were traced using the segmented line tool and their length measured (Fig. 1E and J).

Additionally, after manually tracing cell boundaries using the polygon selection tool, the total area (including nuclei) of neuronal and non-neuronal cells generated by treatment with AraC for 6 days was measured using the grayscale-converted images corresponding to the β-III tubulin channel (schematically illustrated in Fig. 1F). The data from this analysis are shown in González-Burguera et al. (2016, Fig. 3) [1] and served to assess whether phenotypes classified as neuronal and non-neuronal after AraC-induced differentiation were morphometrically distinguishable.

### 2.3. Statistical analysis

All data represent average values obtained from 99 cells in 3 independent experiments (33 cells/experiment). Results were statistically analyzed in GraphPad Prism (version 5.0, GraphPad Software Inc., San Diego, CA) and presented as mean±SEM. Statistically significant differences between two groups were assessed by two-tailed unpaired t-test. *p*<0.05 was considered significant.
