# Supplementary material for: Data for the morphometric characterization of NT2-derived postmitotic neurons
Source: Data Brief. 2016 Apr 13;7:1349–54. doi: 10.1016/j.dib.2016.04.021 (PMC4845154; doi:10.1016/j.dib.2016.04.021)
Supplement: Supplementary file 1 — Supplementary material [file mmc1.pdf]

Advertencia: este formulario no es compatible con la versiÃ³n actual de Acrobat o Adobe Reader.

Actualice el programa a la Ãºltima versiÃ³n para una compatibilidad total.
